# Nystagmus During a Vertigo Crisis in Menière’s Disease—Direction of Nystagmus, Contribution of a Mobile Phone and Therapeutic Implications

**DOI:** 10.3390/jcm13247555

**Published:** 2024-12-12

**Authors:** Valéria Ionescu, Tamadhor Alzarqaa, Saad Albalawi, Yann Lelonge, Pierre Reynard, Alexandre Karkas, Pierre Bertholon

**Affiliations:** 1 Département d’ORL, Centre Hospitalier Universitaire de Saint Etienne, 42055 Saint-Etienne, Franceyann.lelonge@univ-st-etienne.fr (Y.L.); alexandre.karkas@univ-st-etienne.fr (A.K.); 2 Department of Audiology and Neurotology, Hospices Civils de Lyon, 69003 Lyon, France

**Keywords:** Menière’s disease, delayed endolymphatic hydrops, vertical nystagmus, direction changing nystagmus, mobile phone

## Abstract

**Background/Objectives**: Spontaneous nystagmus during vertigo attacks of Menière’s disease has been essentially described as horizontal, beating ipsilaterally (irritative type) or contralaterally (deficit type) to the hearing loss. Our main objective was to describe the characteristics of nystagmus during vertigo attacks. The second objective was to determine the feasibility of self-video recording of eye movements by a mobile phone. The third objective was to discuss the therapeutic implications of the observed nystagmus. **Methods:** We selected patients with definite Menière’s disease according to the Barany Society. Patients were video-recorded during their attacks by videonystagmoscopy (by the physician) and/or mobile phone (by the patient or immediate surroundings). **Results:** Seventeen patients were video-recorded by mobile phone (n = 8) or videonystagmoscopy (n = 8) or both (n = 1). The nystagmus was horizontal in 14 patients of the irritative type (n = 7) and of the deficit type (n = 5) or changed from the deficit to the irritative type (n = 2). The nystagmus was vertical in three patients, either down-beating (n = 2) or up-beating (n = 1). This vertical nystagmus changed to a more classical horizontal nystagmus in two patients. **Conclusions:** The direction of the nystagmus was variable and mostly horizontal, although it could be vertical and could change direction. Thus, the direction of the nystagmus has no value in deducing the affected side of Menière’s disease, which essentially relies on hearing dysfunction. The nystagmus could be video-recorded by a mobile phone, which was objective proof of the impact on daily life. This was a helpful therapeutic aid, particularly when chemical labyrinthectomy was considered.

## 1. Introduction

In 1981, McClure et al. performed electronystagmographic recordings during vertigo crises in eight patients suffering from Menière’s disease (MD) [1]. They demonstrated horizontal nystagmus that was initially of the deficit type (beating on the other side of the hearing loss) then becoming irritative (beating on the side of the hearing loss) with up to three changes in direction during a given episode [1]. In 1986, Nishikawa et al. used a newly devised long recording portable electronystagmograph that enabled recording before the sensation of vertigo and observed that the initial horizontal nystagmus was of the irritative type, changing to a deficit type in one patient [2]. In 1991, Bance et al. observed the same features using electronystagmography in two patients with a change in the direction of nystagmus that may occur within 20 s [3]. An irritative horizontal nystagmus that was not changing was observed using electronystagmography in two patients with MD [4]. In 2009, Kumagami et al., using an infrared video camera, observed 22 patients during Meniere’s crisis that all showed a horizontal nystagmus [5]. In 2018, Reynard et al., using videonystagmoscopy (VNS), were the first to observe an initial vertical nystagmus that was up-beating with a slight torsional component in a patient suffering from a variant of MD named delayed endolymphatic hydrops [6]. The nystagmus secondarily became horizontal–torsional beating contralaterally to the side of the hearing loss [6]. The occurrence of vertical down-beating nystagmus has been recently reported to occur in three out of forty-three patients (7%) during vertigo episodes in MD using a portable recording device [7]. This vertical down-beating nystagmus later changed to a horizontal nystagmus [7].

We herein report the clinical features of seventeen patients suffering from MD who had been video-recorded during their vertigo attacks with VNS (by the physician) and/or mobile phone (by the patient or immediate surroundings). We focalized on the characteristics of nystagmus during vertigo attacks, the contribution of the mobile phone and therapeutic implications of the observed nystagmus.

## 2. Materials and Methods

In this retrospective study, we considered patients with the following:

- A definite MD (‘idiopathic endolymphatic hydrops’) according to the 1995 criteria of the American Academy of Otolaryngology–Head and Neck Surgery (AAO-HNS) [8], recently reviewed by the Barany Society [9]: two or more spontaneous episodes of vertigo each lasting 20 min to 12 h, with fluctuating aural symptoms in the affected ear and audiometrically documented hearing loss on at least one occasion. We included patients with recurrent attacks of vertigo occurring many years after hearing loss, at least profound, which is a variant of MD that could be either called ‘delayed endolymphatic hydrops’ (DEH) or ‘delayed MD’ [6,9]. Patients with bilateral MD were excluded.
- At least one video recording of eye movements during a vertigo attack. This video recording was obtained in our clinic by examination of the nystagmus under VNS and/or outside of our clinic by using the mobile phone of the patient.

All patients underwent a detailed interview and had a complete otoneurological examination. Pure tone and speech audiometry, video head impulse test (vHIT), videonystagmogaphy (VNG) and cervical vestibular evoked myogenic potentials (cVEMPs) were performed and repeated according to clinical findings. All patients had brain magnetic resonance imaging (MRI) and a complementary computed tomography (CT) of the temporal bone according to clinical findings.

Treatment of MD usually includes long-term oral betahistine (48 mg/day) plus acetyleucine (2 g/day) with a salt-restricted diet and limitation of alcohol and caffeine consumption. Flunarizine (5 to 10 mg daily) could be proposed for a limited period of 6 months in case of associated migraine. Daily physical activity was encouraged, and vestibular rehabilitation was often proposed in older patients who maintained chronic instability after vertiginous attacks. Appropriate psychological support was regularly offered due to the emotional triggers of a crisis. The latter psychological factor associated with MD prompted us to record the nystagmus during a crisis in order to obtain objective proof of the impact of vertigo on daily life, particularly when chemical labyrinthectomy was being considered. Chemical labyrinthectomy was usually proposed in patients with an average pure tone hearing loss of more than 60 dB who complained of incapacitant vertigo attacks occurring with a frequency of at least one episode per month for at least 6 months [10]. Due to this frequency, it was easy to ask patients to video-record his/her next crisis with a mobile phone. An alternative and rarer indication for chemical labyrinthectomy was the occurrence of drop attacks due to the risk of traumatism during the fall [11]. Chemical labyrinthectomy was performed using a titration technique. Microscopic examination of the tympanic membrane was followed by topical anesthesia by application of cotton soaked with a medicated cream of lidocaine–prilocaine on the whole eardrum for one hour. After fitting a slightly downward-folded spinal needle with an insulin syringe, approximately 0.5 mL of gentamycin sulfate solution 40 mg/mL (not buffered with sodium bicarbonate) was slowly injected through the posterior–inferior quadrant of the tympanic membrane (facing the round window area) into the middle ear. The patient remained supine with the head rotated slightly to the untreated side for one hour and was told not to swallow. The decision to perform additional trans-tympanic gentamycin injections, with an interval of one month between each injection, was based on the degree of vestibular symptom control after complete audio-vestibular re-assessment [6,10,11,12,13].

Due to recent Guidelines of the French Otorhinolaryngology–Head and Neck Surgery Society, we tend to offer dexamethasone trans-tympanic injection (4 mg/mL) before chemical labyrinthectomy, as it may be efficient on vertigo episodes with a low risk of hearing loss aggravation [14]. It is technically performed in the same way as chemical labyrinthectomy, apart from replacing gentamycin with dexamethasone.

All these different therapeutic options were always explained and discussed in a personalized approach with the patient in order to obtain joint decision-making.

This study was carried out at Saint Etienne University Hospital over a period of 20 years. The first patients were video-recorded with VNS by chance during a vertigo episode occurring in consultation, but all mobile phone recordings have been performed over the last 2 years.

In accordance with French regulations, a specific signed agreement was obtained for the 3 detailed video-recorded patients, but formal institutional review board approval was not required for a retrospective study of this type.

## 3. Results

### 3.1. Study Results

We report the clinical features of seventeen patients, twelve with MD and five patients with DEH. The mean age was 61 years [range 29–85 years], with eleven men (mean age 60 years [range 35–85]) and six women (mean age 62 [range 29–83]).

A previous history of migraine was observed in eight patients. One patient had a right cerebellar infarction on brain MRI that could not be responsible for recurrent vertigo episodes (see Table 1, patient n°7). Two patients had severe vascular risk factors (patient n°11, detailed below, and n°13). One patient had a previous middle ear surgical intervention during infancy (patient n°12). Fluctuating mild-to-severe ipsilateral hearing loss (HL) was observed in patients with MD and profound or complete HL in DEH.

By definition, patients complained of at least two vertigo crises, but all had many more episodes. The nystagmus during the vertigo crises was video-recorded in our clinic by VNS in eight patients, by mobile phone in eight patients, or both in one patient (see patient n°11 detailed below). Horizontal nystagmus was observed in fourteen patients (see Table 1), seven patients with irritative type nystagmus, five with the deficit type, and two patients with direction changing nystagmus from deficit to irritative type (patients n°5 and 11 detailed below). Vertical nystagmus during the vertigo was observed in three patients (see Table 2), up-beating in one and down-beating in two. The patient with the up-beating nystagmus is a woman, now aged 29, suffering from left DEH (patient n°15) [6]. During a one-hour vertigo crisis, VNS demonstrated a vertical up-beating with a torsional component to the right shoulder, which secondarily became right horizontal torsional, i.e., deficit type (patient already published with accessible video, see [6]). One of the patients with down-beating nystagmus is a man, now aged 75, suffering from left MD (patient n°16). At the age of 63, he had a drop attack, followed by vertigo, which lasted 3 h. During the vertigo episode, the observed spontaneous nystagmus was essentially down-beating with a torsional component to the left shoulder that changed to right horizontal–torsional nystagmus, i.e., deficit type, and later regained the same down-beating with a torsional component to the left shoulder as initially observed (patient already published with accessible video, see [11]). The other patient with down-beating nystagmus is a 58-year-old man with left MD who had no change of his vertical nystagmus during a one-hour vertigo crisis, despite five video recordings (see patient n°17 detailed below). All these three patients with vertical nystagmus were seen in our clinic at the very onset of their vertigo episodes.

Eight patients in this series could be managed with oral treatments and sometimes with psychological support and/or physiotherapy, although one is under discussion for dexamethasone or gentamycin injection (patient n°13). A chemical labyrinthectomy by gentamycin injection was administered in nine patients. Of note, this treatment had to be performed after the inefficiency of three dexamethasone injections in one patient (see patient n°11 detailed below). Chemical labyrinthectomy was efficient in eight out of nine patients, with a mean follow-up of 5.6 years. Of note, one patient for whom we considered that chemical labyrinthectomy was initially efficient needed a new gentamycin re-injection 2 years after a previous one (see Table 2, patient n°15). There was an inefficiency of gentamycin injection in one patient (out of nine patients), in the context of DEH with complete HL, who secondarily had a surgical labyrinthectomy (see Table 1, patient n°12).

### 3.2. Case Series

The following three case histories illustrate representative patients:

#### 3.2.1. Patient n°10

A 63-year-old man with no previous medical history (including no migraine) complained of a first vertigo episode at the age of 33 years. At the age of 37, he suffered from sudden left hearing loss without vertigo. A pure-tone audiometry confirmed a moderate-to-severe sensorineural hearing loss (SNHL) on the left side. Brain MRI was normal. At the age of 59, he complained of a second vertigo attack lasting 4 h with vomiting, leading to the diagnosis of left MD. He experienced a recurrence of vertigo attacks at a frequency of approximately once a month for 2 years (between the ages of 60 and 62 years). The audiometry revealed a profound SNHL on the left side and a high-frequency SNHL on the right side. There was a 78% deficit of the left ear at caloric stimulation. vHIT showed normal responses on both sides. cVEMPs were absent on the left side, with normal responses on the right side. A new MRI of internal auditory canals showed no lesion. The patient was asked to video-record his eyes during a vertigo crisis, and right horizontal nystagmus (deficit type) was obvious (see Video S1). Due to disabling recurrent vertigo crises and left profound SNHL, one trans-tympanic gentamycin injection was performed on the left side. Four days later, the patient complained of disequilibrium, with a 93% deficit of the left ear (caloric stimulation) and a gain of 0.3 for the left horizontal semi-circular canal (vHIT). There was no recurrence of vertigo attacks at the 1-year follow-up.

To summarize, the patient had left MD. Mobile phone recording of the nystagmus during vertigo spells confirmed right horizontal nystagmus (deficit type). Chemical left labyrinthectomy (one injection) was effective on follow-up at one year.

#### 3.2.2. Patient n°11

A 66-year-old woman complained of a long history of vertigo attacks starting at the age of 30 years. Vertigo episodes lasted hours with vomiting and right tinnitus, and some episodes had been associated with headaches. At age 58, pure-tone audiometry revealed a moderate SNHL on the right side. Brain MRI was normal. She had a previous history of cardiac arrhythmia in the context of a familial long QT syndrome (abnormality of gene SCN5A, which is different from the abnormality observed in Jervell and Lange Nielsen syndrome), for which a pacemaker was implanted (precluding new MRI). She had migraines. At the age of 65 years, she described a reactivation of vertigo attacks despite oral treatment, including flunarizine. Pure-tone audiometry fluctuated from moderate to severe SNHL on the right side. vHIT showed normal responses on both sides. cVEMPs were absent on both sides. The patient was asked to video-record her eyes, and she rapidly provided two videos of her left eye in two different vertigo crises showing left horizontal nystagmus (see Video S2). She had two trans-tympanic dexamethasone injections with the improvement of the vertigo attacks, although the injections were performed concurrently with the diagnosis and medical care for sleep apnea (she was equipped with a C-PAP). Three months later, she complained of four drop attacks during a one-month period, without loss of consciousness or cardiac arrhythmia, and no trauma during the fall but vertiginous sensation when she was on the ground. A new (third) dexamethasone trans-tympanic injection was performed, but it was inefficient as the patient reported vertigo episodes every day and was homebound. When re-assessing the patient 2 weeks later in our clinic, we observed (under VNS) an initial left horizontal nystagmus (see Video S3) that changed one hour later to right horizontal nystagmus (see Video S4). The vHIT gain for the right horizontal canal during the crisis was pathological (0.24) and almost normalized at the end of the crisis (0.71). We performed, on the same day, a right trans-tympanic injection of gentamycin. A few days after the injection, she complained of permanent dizziness/instability with no recurrence of vertigo episodes or drop attacks at the 2-month follow-up. The gain on vHIT gain was decreased for all three semi-circular canals on the right side (around 0.2 for the horizontal canal). Instability improved by physiotherapy.

To summarize, the patient had right MD. Two mobile phone recordings of the nystagmus during two different vertigo spells confirmed left horizontal nystagmus (deficit type). The VNS recording during the vertigo episode in our clinic showed left horizontal nystagmus that changed to right horizontal nystagmus during the same crisis. MD was complicated by four drop attacks. One trans-tympanic gentamicin injection was performed after the inefficacy of three trans-tympanic dexamethasone injections. The patient had instability but no recurrence of vertigo attacks or drop attacks at the 2-month follow-up.

#### 3.2.3. Patient n°17

A 57-year-old man complained of left tinnitus starting at the age of 44 years without vertigo and with normal auditory results. There was no history of migraine. At the age of 51 years, he experienced four vertigo attacks during a one-year period, with vomiting, left hearing loss and tinnitus. One of these vertigo episodes started in our clinic and lasted one hour. VNS revealed a vertical down-beating nystagmus with a slight torsional component to the right shoulder, and this nystagmus remained identical in five video recordings (see Video S5). We were able to test his postural deviation, which was backward with eyes closed, reproducing a peripheral vestibular deficit in the vertical plane (vertical down-beating nystagmus). Audiometry revealed a predominantly low-frequency moderate SNHL on the left side (60–70 dB) and normal hearing on the right side. There was a 53% deficit of the left ear on caloric stimulation, normal bilateral cVEMP responses and normal vHIT test for all semi-circular canals. A brain MRI showed no pathological findings. Although the patient had no recurrence of vertigo attacks at the 6-year follow-up, he complained of disabling tinnitus and hearing loss on the left side, partly ameliorated by oral medication, hearing aids and various psychological supports. He developed no neurological pathology at the 6-year follow-up.

To summarize, the patient had left MD. Five VNS recordings during a one-hour vertigo episode in our clinic showed a vertical down-beating nystagmus with a slight torsional component to the right shoulder that did not change. No recurrence of vertigo attacks at the 6-year follow-up.

## 4. Discussion

We would like to discuss the characteristics of nystagmus during vertigo attacks, the feasibility of self-video-recording eye movements by a mobile phone and the therapeutic implications of the observed nystagmus.

### 4.1. The Characteristics of Nystagmus During Vertigo Attacks

The nystagmus appears to be highly variable in this series, beating initially horizontally in fourteen patients and vertically in three other patients (see Table 3). The horizontal nystagmus was of the irritative type in seven patients, of the deficit type in five patients, and changing from the deficit to the irritative type in two other patients. The occurrence of horizontal nystagmus has been well-documented since the article by McClure et al. in 1981 [1]. There can be up to three changes in the horizontal nystagmus during a given episode [1,15]. The main practical consequence is that the direction of the nystagmus is of no value to deducing the affected side in MD or DEH, which essentially relies on hearing dysfunction. Of note, our recording of horizontal nystagmus was mostly performed by mobile phone and often delayed regarding the onset of the vertigo episode, giving no argument on the direction of the initial horizontal nystagmus. Nishikawa et al., using a long recording (1 day) portable electronystagmograph that enabled the recording of eye movements before the sensation of vertigo, observed that the initial horizontal nystagmus was of the irritative type, changing to a deficit type in one patient [2]. However, Phillips et al., using an even longer (30 days) electrooculography recording, observed that the initial nystagmus was of the deficit type, changing to an irritative type [15]. The question of the direction of the initial horizontal nystagmus remains unsolved, and our series even raises the question of an initial nystagmus being vertical. Indeed, the three patients with vertical nystagmus all started their vertigo episodes in our consultation, and we were able to observe and video-record their vertical nystagmus at the very onset of the vertigo episode. However, we cannot definitively ascertain that the initial nystagmus was vertical for two reasons. Firstly, the observation of nystagmus can precede the sensation of vertigo [2,15]. Secondly, a change in the direction of the nystagmus can occur within 30 s and even 20 s [3,15]. In our series, the vertical nystagmus was down-beating in two patients and up-beating in one [6,11]. Such vertical nystagmus in the primary position of gaze is generally indicative of central nervous system pathology [16]. However, the occurrence of vertical down-beating nystagmus is not that rare in vertigo attacks of MD, as it has been recently reported to occur in three out of forty-three patients (7%) using a portable recording device [7]. Vertical up-beating nystagmus has also been reported to occur in one out of seventy patients (1.4%) during vertigo episodes of MD [17]. Thus, patients in our series and the recent literature definitively confirm that the nystagmus observed during a crisis of MD can have a vertical direction, either down- or up-beating (with a slight torsional component) [6,7,11,17,18]. However, Young et al. compared patients with MD and those suffering from vestibular migraine (VM) and demonstrated that the occurrence of vertical nystagmus is less frequent in MD than in VM [7,17,19]. Interestingly, they demonstrated that the velocity of the ictal nystagmus (independently of its direction) is higher in MD than in VM [7,17,19]. In our series, the vertical nystagmus converted into a more classical horizontal–torsional component of the deficit type in two patients (see Table 2, patients n°15 and 16) and even regained the previous vertical direction in one of them (see Table 2, patient n°16) [6,11]. The same phenomenon was observed in the MD series of Young et al. as the torsional down-beating nystagmus observed in three patients was followed by contraversive horizontal nystagmus [7]. Although a change in the direction of the nystagmus has been reported in VM, this phenomenon is rare and strongly supports the diagnosis of MD versus VM [7,19]. More challenging is our patient with down-beating nystagmus (with a slight rotatory component), as there was no change in the direction of the nystagmus despite five video recordings during a one-hour vertigo episode (patient n°17 detailed above). This patient had no previous history of migraine and developed no other neurological pathology despite 6 years of follow-up. Although the recording was sequential, this patient raises the question of a non-changing vertical nystagmus that could be related to MD. Answering this question requires further observations of vertical non-changing nystagmus with continuous recording.

### 4.2. The Feasibility of Self-Video-Recording Eye Movements by a Mobile Phone

In this series, the nystagmus was video-recorded by VNS in our clinic in eight patients, by mobile phone in eight patients, or both in one patient. In the latter patient for whom we have the VNS and mobile phone recording, we can compare and conclude on the feasibility of mobile phone recording (see patient n°11 above). Indeed, twenty-six patients in a series of thirty patients with MD were able to send satisfactory eye movement videos, with horizontal nystagmus in twenty-one patients and no nystagmus in five patients [20]. Of the latter five patients, three were finally diagnosed with migraine and two with anxiety disorders [20]. The main limitation of mobile phone videos is that the recording is performed at ocular fixation, which diminishes the intensity of peripheral vestibular nystagmus. Consequently, the observation of nystagmus with a mobile phone has a high positive diagnostic value for disabling vertigo in the context of MD. However, the absence of nystagmus can be related to false negative recording due to inhibition by fixation. In the latter circumstance, it will be interesting to use a specific video-oculography portable device [7], the CAVA system (Continuous Ambulatory vestibular Assessment), which is a near-continuous electronystagmography monitoring device that can be used with eyes closed [15,21], or the Nystagmocatcher (Vertigocatcher Diagnostics AB, Stockholm, Sweden) [18]. The latter has the advantage of being a commercially available and relatively inexpensive smartphone-based adapter [18]. The adapter consists of a foam ring that is attached to the lens of any mobile phone and held on the eye that should be recorded while the other eye is patched to prevent light from outside [18]. The recording is not continuous, but the patient can be asked to use the device every 15 min from the onset of the attack until the end [18].

### 4.3. The Therapeutic Implications of the Observed Nystagmus

Self-description of a vertigo crisis by the patient is subjective by nature, and this lack of objectivity is amplified in vertigo due to MD or DEH, where psychological factors play an important role in generating the disease and/or triggering vertigo crises [22,23]. Further, the possibility of co-existing vestibular disorders, either primary or secondary, is not rare [24,25]. For example, MD or DEH can be associated with anxiety disorders, such as panic attacks. In the latter, patients complain of dizziness, lightheadedness and unsteadiness, which are the second most common physical manifestations after cardiopulmonary symptoms (chest pain, palpitation and dyspnea), but vestibular/spinning sensation is usually less intense in a panic attack than in a structural lesion of the labyrinth [25]. In this series and by definition, patients with MD or DEH complained of vertigo of more than 20 min, but a subset of patients had several vertigo crises per month or even per week. Thus, it was easy to ask the patient to video-record his/her next crisis with a mobile phone. The observation of obvious nystagmus was objective proof of the disability and a helpful therapeutic aid in proposing a chemical labyrinthectomy. The pre-requisite for chemical labyrinthectomy was a frequency of at least one vertigo episode for at least 6 months (despite treatment) and an average pure tone hearing loss of more than 60 dB. The chemical labyrinthectomy was performed using a titration technique, i.e., repeating the gentamycin injection every month without trying to obtain complete deafferentation [6,10,11,12,13]. This restricted indication, guided by the observation of nystagmus during a crisis, is probably the reason for the efficacy of chemical labyrinthectomy in eight out of nine patients (see Table 3). However, the follow-up of these eight patients is limited to 5.6 years, and a subset of patients will probably need re-injection, as was already the case in one woman (case report n°16). Chemical labyrinthectomy (despite two gentamycin injections) was inefficient in one out of nine patients who had recurrent vertigo and no deafferentation on vestibular testing, but this was probably related to previous middle ear surgery in infancy (patient n°12). Indeed, it has been demonstrated that intratympanic gentamycin was significantly less effective in controlling vertigo in patients who had previous otologic surgery, possibly due to bone dust causing adhesions in the area of the round window membrane or annular ligament of the stapes that impeded the entry of gentamycin into the inner ear [26]. As this patient had complete hearing loss in the context of DEH, he was cured of recurrent vertigo after surgical labyrinthectomy (patient n°12). More challenging is our attitude when no nystagmus can be recorded by mobile phone during vertigo attacks. No conclusion can be ascertained, as the absence of nystagmus can be related to false negative results due to ocular fixation. Thus, the diagnosis can be an episode of vertigo in MD, a vestibular migraine (the velocity of the ictal nystagmus is lower in VM than in MD), a panic attack, a combination of vestibular disorders, and even a malingerer could not be excluded. In this circumstance, we were reluctant to propose a chemical labyrinthectomy, and an alternative option, such as trans-tympanic dexamethasone injection, was discussed on a case-by-case basis. Interestingly, new devices such as the CAVA or the Nystagmocatcher may optimize the diagnosis and medical care for MD, panic attacks and malingerer [18,21]

Although we wanted to share the simplicity of the use of a mobile phone and its effectiveness, there are several limitations in this study already emphasized above. These limitations include the retrospective design of the study, the small number of participants and the one-shot mobile phone recording, which is performed at fixation. Further, although we had a good effect of chemical labyrinthectomy, the follow-up is limited, and gentamycin re-injection will probably be necessary in some patients.

## 5. Conclusions

The direction of the nystagmus during vertigo attacks in MD is mostly horizontal, although it can be vertical and can change direction. From a diagnostic point of view, the direction of the nystagmus is of no value to deducing the affected side in MD. The nystagmus during vertigo attacks can be video-recorded by the mobile phone of the patient, which is objective proof of the impact on daily life and confirms a disabling MD. From a therapeutic point of view, this was a helpful aid when chemical labyrinthectomy was being considered.

## Figures and Tables

**Table 1:** Clinical features of the patients with horizontal nystagmus during vertigo crisis (vHIT—video head impulse test (only before gentamycin injection); HL—hearing loss (only before gentamycin injection); NP—not performed; cVEMP—cervical vestibular evoked myogenic potential; CP—canal paresis; N—normal; VNS—videonystagmoscopy).

Patient Sex, Age	History	Diagnosis	Audiometry	Nystagmus Recording	VNS or Phone	Canal Paresis (CP), cVEMP (Threshold Right/Left), vHIT	Imaging	Treatment -Follow-Up (Period with No Vertigo)
No 1 M, 41	Migraine +	Right MD	Right severe HL Left Normal	Right horizontal nystagmus (irritative)	VNS	Right CP (47%) cVEMP = N vHIT = NP	N	Gentamycin injection (10) -Favorable (17 y)
No 2 M, 85	Migraine +	Left DEH	Left profound HL Right moderate HL	Left horizontal nystagmus (irritative)	VNS	Caloric tes t = N cVEMP = absent on left side vHIT = N	N	Gentamycin injection (2) -Favorable (1 y)
No 3 M, 60	Migraine −	Right DEH	Right total HL Left Normal	Right horizontal nystagmus (irritative)	VNS	Normal caloric responses cVEMP = present 100/90 vHIT = NP	N	Gentamycin injection (2) -Favorable (1 y)
No 4 M, 79	Migraine +	Right DEH	Right total HL Left N	Left horizontal nystagmus (deficit)	VNS	Right CP (84%) cVEMP = present 95/85 vHIT = NP	N	Gentamycin injection (2) -Favorable (9 y)
No 5 F, 83	Migraine +	Left MD	Left severe HL Right Mild HL	Right horizontal nystagmus (deficit), then left horizontal nystagmus (irritative)	VNS	Left CP (86%) cVEMP = present 100/100 vHIT = NP	N	Medical treatment -Favorable (15 y)
No 6 M, 37	Migraine +	Left MD	Left moderate HL Right Normal	Left horizontal nystagmus (irritative)	Mobile phone	Left CP (52%) cVEMP = present 105/110 vHIT = N	N	Medical treatment -Favorable (1 y)
No 7 F, 63	Right cerebellar infarct Migraine +	Right MD	Right severe HL Left Normal	Right horizontal nystagmus (irritative)	Mobile phone	Right CP (62%) cVEMP = NP vHIT = N	Right cerebellar infarct	Medical treatment -Favorable (10 m)
No 8 F, 58	Migraine +	Right MD	Right moderate HL Left Mild HL	Right horizontal nystagmus (irritative)	Mobile phone	Caloric test NP cVEMP = present 120/100 vHIT = N	N	Medical treatment -Favorable (13 m)
No 9 M, 65	Migraine −	Right MD	Right severe HL Left Normal	Right horizontal nystagmus (irritative)	Mobile phone	Right CP (19%) cVEMP = absent on the right side/left 105 vHIT = N	N	Medical treatment -Favorable (1 y)
No 10 M, 63	Migraine −	Left MD	Left profound HL Right mild HL	Right horizontal nystagmus (deficit)	Mobile phone	Left CP (78%) cVEMP = absent on the left side/right 100 vHIT = N	N	Gentamycin injection (1) -Favorable (1 y)
No 11 F, 66	Migraine − Long QT interval of genetic origin Pacemaker	Right MD	Right severe HL Left Mild HL	Left horizontal nystagmus (deficit), then right horizontal (irritative)	Mobile phone (x2) VNS	Right CP (30%) cVEMP absent bilateral vHIT = Normal	N	Medical treatment Dexamethasone inject (3), then gentamycin injection (1) -Favorable (2 m)
No 12 M, 35	Right tympanoplasty (at the age of 12) Migraine −	Right DEH	Right total HL Left N	Left horizontal nystagmus (deficit)	Mobile phone	Left CP (60%) cVEMP = NP vHIT = N	N	Gentamycin injection (2), then surgical labyrinthectomy -Favorable (6 m)
No 13 F, 75	Migraine + Vascular risk factors	Left MD	Left severe HL Right mild HL	Left horizontal nystagmus (deficit)	Mobile phone	Right CP (51%) cVEMP = NP vHIT = N	N	Medical treatment -Not favorable (discussion over dexamethasone or gentamycin injection ?)
No 14 M, 69	Migraine –	Right MD	Right severe HL Left mild HL	Left horizontal nystagmus (deficit)	Mobile phone	Right CP (68%) cVEMP = absent bilateral vHIT = N	N	Medical treatment -Favorable (4 m)

**Table 2:** Clinical features of the patients with vertical nystagmus during a vertigo crisis. Complementary exploration results before any possible trans-tympanic injection. MD, Meniere’s disease; DEH, delayed endolymphatic hydrops; HL, hearing loss; VNS, videonystagmoscopy; CP, canal paresis; vHIT, video head impulse test; cVEMP cervical vestibular evoked myogenic potential; N, normal; NP, not performed.

Patient Sex, Age	History	Diagnosis	Audiometry	Nystagmus Recording	VNS or Phone	Canal Paresis (CP), cVEMP, vHIT	Imaging	Treatment Follow-Up (Period with No Vertigo)
No 15 F, 29	Migraine −	Left DEH	Left total HL Right N	-1 h vertigo -Vertical up-beating with a torsional component to the right shoulder, then right horizontal nystagmus (deficit type)	VNS	Normal caloric responses cVEMP = threshold 100/125 vHIT = NP	N	Gentamycin injection (1) Favorable (2 y) After 2 years, recurrence of three vertigo episodes, less severe. One new gentamycin injection (1) Favorable (6 y)
No 16 M, 75	Migraine −	Left MD	Left severe HL Right N	-Drop attack in the waiting room, followed by a 3 h vertigo -A few minutes after the fall, vertical down-beating nystagmus with a torsional component to the left shoulder, then right horizontal nystagmus, then regain the same down-beating nystagmus with a torsional component to the left shoulder	VNS	Left CP (61%) cVEMP = threshold 100/110 vHIT = NP	N	Gentamycin injection (6) Favorable (10 y)
No 17 M, 58	Migraine −	Left MD	Left moderate HL Right N	-1 h vertigo -Vertical down-beating nystagmus with a slight torsional component to the right shoulder (five video recordings)	VNS	Left CP (53%) cVEMP = NP vHIT = Normal	N	Medical treatment Favorable for vertigo (6 y) Disabling tinnitus and hearing loss

**Table 3:** Summary of the characteristics of the nystagmus in 17 patients and efficacy of chemical labyrinthectomy (9 patients).

Nystagmus Characteristics				Chemical (Gentamycin) Labyrinthectomy		
Direction	Number	Type of Initial Nystagmus	Change	Number	Efficacy	Additional Surgical Treatment to Initial Gentamycin Injection
Horizontal	14 (14/17 = 82%)	Deficit nystagmus = 7 Irritative nystagmus = 7	One change in two patients (deficit to irritative type) (patients 5 and 11)	7	6/7	1/7 that needed surgical labyrinthectomy (patient 12)
Vertical	3 (3/17 ( = 18%)	Up-beating nystagmus = 1 Down-beating nystagmus = 2	One change (one patient) = up-beating becoming horizontal = deficit type (patient 15) Two changes (one patient) = down-beating becoming horizontal (deficit type), then regaining the same down-beating component (patient 16)	2	2/2 but gentamycin re-injection after 2 years (patient 15)	

## Data Availability

The data presented in this study are available upon request from the corresponding author. The data are not publicly available due to ethical, legal and privacy issues.

## Supplementary Materials

The following supporting information can be downloaded at https://www.mdpi.com/article/10.3390/jcm13247555/s1, Video S1: (patient n°10): obvious right horizontal nystagmus with a mobile phone during a vertigo crisis in left MD; Video S2 (patient n°11): obvious left horizontal nystagmus on the left eye with a mobile phone during a vertigo crisis in right MD; Video S3 (patient n°11): left horizontal nystagmus with VNS during a vertigo crisis in right MD; Video S4 (patient n°11): right horizontal nystagmus with VNS one hour later (same vertigo crisis) in right MD; Video S5 (patient n°17): essentially down-beating nystagmus with VNS that did not change despite five video recordings during a one-hour vertigo crisis in left MD.
